# Trait Associations across Evolutionary Time within a *Drosophila* Phylogeny: Correlated Selection or Genetic Constraint?

**DOI:** 10.1371/journal.pone.0072072

**Published:** 2013-08-28

**Authors:** Vanessa Kellermann, Johannes Overgaard, Volker Loeschcke, Torsten Nygaard Kristensen, Ary A. Hoffmann

**Affiliations:** 1 Department of Bioscience, Aarhus University, Aarhus, Denmark; 2 Department of Biological Sciences, Monash University, Victoria, Australia; 3 Department of Molecular Biology and Genetics, Aarhus University, Tjele, Denmark; 4 NordGen - Nordic Genetic Resource Center, Ås, Norway; 5 Department of Genetics, Bio21 Institute, The University of Melbourne, Victoria, Australia; Fred Hutchinson Cancer Research Center, United States of America

## Abstract

Traits do not evolve independently. To understand how trait changes under selection might constrain adaptive changes, phenotypic and genetic correlations are typically considered within species, but these capture constraints across a few generations rather than evolutionary time. For longer-term constraints, comparisons are needed across species but associations may arise because of correlated selection pressures rather than genetic interactions. Implementing a unique approach, we use known patterns of selection to separate likely trait correlations arising due to correlated selection from those reflecting genetic constraints. We examined the evolution of stress resistance in >90 *Drosophila* species adapted to a range of environments, while controlling for phylogeny. Initially we examined the role of climate and phylogeny in shaping the evolution of starvation and body size, two traits previously not examined in this context. Following correction for phylogeny only a weak relationship between climate and starvation resistance was detected, while all of the variation in the relationship between body size and climate could be attributed to phylogeny. Species were divided into three environmental groups (hot and dry, hot and wet, cold) with the expectation that, if genetic correlations underpin trait correlations, these would persist irrespective of the environment, whereas selection-driven evolution should produce correlations dependent on the environment. We found positive associations between most traits in hot and dry environments coupled with high trait means. In contrast few trait correlations were observed in hot/wet and cold environments. These results suggest trait associations are primarily driven by correlated selection rather than genetic interactions, highlighting that such interactions are unlikely to limit evolution of stress resistance.

## Introduction

The distribution and abundance of many ectothermic animals including insects depends on adaptations to extremes of environmental temperature, humidity and food resources [1]–[6]. It is therefore important to understand the factors that have shaped the evolution of environmental tolerance, particularly when predicting future distributions and population dynamics. Evolutionary trajectories under climate change may in part be driven by genetic interactions among traits [7], [8]. Even in the presence of ample genetic variation for key traits, limits may arise due to the non-independent evolution of traits [9]. For instance, if heat and cold resistance in ectotherms show a trade-off and there is selection under climate change for tolerating both extremes, directional adaptation to climate change will be difficult.

*Drosophila* species have frequently been used as a model to dissect the importance of genetic interactions in shaping trait evolution [5], [10]–[14]. At the intra-specific level, experiments have provided evidence for trade-offs between heat and cold resistance as well as starvation and cold resistance, particularly in *Drosophila melanogaster* [10], [15], [16]. Although these experiments provide data on short term responses within populations, they may not be representative for evolutionary interactions that have been under selection over a longer (evolutionary) timescale. Thus, if trait correlations play an important role in shaping ecological adaptation, we would anticipate a signature of trait associations to persist at the species level. Species comparisons have shown that in some cases correlated trait evolution can be found [17] whereas in other cases it appears absent [18]. In addition, correlations may depend on phylogenetic relatedness [19]. This has previously been difficult to investigate because such an analysis requires a large number of species with known phylogeny that are tested under comparable conditions.

Trait associations may arise for a number of reasons. Firstly associations may be caused by similar genes and mechanisms underlying specific sets of traits (genetic interactions), secondly associations may arise because species that share a recent evolutionary history also share similar trait values (phylogenetic relatedness) and finally correlations may occur because specific environments select for multiple traits at the same time (correlated selection). In the first two cases, the evolution of different traits will not be independent and the direction of evolution may be constrained [9], [20]. In the last case, the traits are free to evolve in any direction with correlated selection pressures driving trait associations.

Here we distinguish between these hypotheses by contrasting situations where correlated selection pressures differ, and then examine trait correlations in these situations after controlling for phylogeny. If consistent patterns are found regardless of whether both traits are under selection, trait associations are likely to reflect constraints at the genetic level. However if trait associations only appear in a predicted direction when there is selection on both traits then correlated selection is the likely explanation for this pattern. We use a common garden approach applied to more than 90 *Drosophila* species to test these hypotheses, combining new measurements of starvation resistance and body size with recently published data for desiccation, heat and cold resistance [4], [21].

Initially we determined the extent to which starvation resistance and body size were driven by climatic variables and phylogeny. We then investigated patterns of correlated evolution between resistance to desiccation, starvation, heat, and cold as well as body size. Correlation patterns of traits as well as variation in size and starvation resistance have not been previously described for this species set. To test the hypothesis that trait correlations reflected genetic interactions rather than correlated selection, we divided species into 3 ecological types (hot and dry, hot and wet, cold) based on their known distributions. We hypothesised that, if genetic interactions drive trait associations, they would persist regardless of the environment; alternatively if trait associations were driven by selection, we would expect trait correlations to differ across environments and trait means to fit with a priori expectations of selection pressures across the environments.

## Materials and Methods

Previous work established a relationship between traits, phylogeny and environment for cold, heat and desiccation resistance [4], [21]. To determine the role of correlated evolution in driving trait evolution for all traits, we extracted the data from these two studies and combined it with unpublished data for starvation resistance and body size. Initially, we examine the relationship between starvation resistance, body size and climate to establish the role of climate in shaping the evolution of these traits. From here we take all the trait data and examine correlated trait evolution, which has previously not been considered.

### Sources and maintenance of experimental animals

Previous studies include detailed information on the sources and maintenance of experimental animals [4], [21]. Briefly *Drosophila* species originated from 5 major sources (field collections from Denmark, field collections from Australia, Jean Robert David, Gif-sur-Yvette, France; the *Drosophila* Species Stock Center, San Diego, USA and the *Drosophila* Genetic Resource Center, Kyoto, Japan) - see Table S1 in [4] for a complete list of species and their respective collection and source information. Where flies were collected from the field they were collected from private property with the permission of the landowners. For each species only one line (i.e one genetic background) was examined.

Prior to testing, the flies were maintained on an oat-based medium (Leeds) at 20°C on a 12∶12 light cycle for a minimum of two generations with population sizes of approximately 200 individuals. Optimum temperature is likely to differ across species, particularly for cold and warm adapted species. To account for this, species were reared at the moderate temperature of 20°C. A number of cactophilic species had special dietary requirements and these species were therefore maintained on a banana-Opuntia medium using the recipe from the *Drosophila* Species Stock Center, San Diego, USA (URL:https://stockcenter.ucsd.edu/info/food_banana_Opuntia.php). These common garden experiments do not address species differences in plastic responses that might be triggered by exposure to different temperatures or other conditions.

#### Estimations of climatic variables

To determine the extent to which starvation resistance and body size were driven by climate, distribution information for each species was compiled from geographical data (GPS coordinates) from published studies for a wide number of *Drosophila* species from the taxodros website (http://www.taxodros.uzh.ch/). Global climate data for each location were obtained from the WorldClim data set (www.worldclim.org) [22]. Duplicate records in terms of species, latitude and longitude were removed. Nine temperature and precipitation variables thought to be related to the evolution of stress resistance were chosen. Four of these, namely (1) annual mean temperature (AMT), (2) absolute maximum temperature of the warmest month (Tmax), (3) absolute minimum temperature of the coldest month (Tmin) and (4) temperature seasonality (Tsea), are the most likely candidates for shaping the evolution of heat and cold resistance. In addition, (5) annual precipitation (Pann), (6) precipitation of the driest month (Pdry), (7) precipitation of the wettest month (Pwet), (8) precipitation seasonality (Psea) and (9) drying power of air (dpa), a measure of aridity [21], are likely to be important for the evolution of desiccation and heat resistance. Preliminary analysis revealed high autocorrelation between AMT and Tmin (r^2^ = 0.94); as Tmin was also highly correlated to Tsea (r^2^ = 0.93), Tmin was removed from further analyses. Environmental variables with r^2^<0.90 were not considered to be highly correlated. Latitude is often used in studies as a proxy for climate and was also included, particularly as it allowed for comparisons to other studies. Climate data was averaged across the distribution of each species. Non phylogenetic least squares regression was then implemented to determine the relationship between stress traits and climate variables. The relationship between stress traits and the climate variables was estimated singularly using the lm() function in R. We also considered a multiple predictor model, beginning with the climate variable with lowest AIC and then adding additional variables based further changes in AIC. However we found a single predictor model to explain the variation in traits better than a multiple predictor model (Table S1). Climate variables with the lowest AIC were used for further phylogenetic analyses. Sampling bias is inherent in these types of datasets, but is unlikely to influence the observed patterns [21].

Using the same environmental data set we then divided species into three categories based on temperature and precipitation. These climatic variables were expected to reflect a range of selection pressures and have previously been shown to be important in driving the evolution of climate related traits in *Drosophila* [4], [21]. Hot environments were defined as those having an annual mean temperature >18°C, these environments were then split into moderate/dry (<1500 mm annual precipitation) and wet (>1500 mm annual precipitation) environments. Environments where annual mean temperature was <18°C were deemed cold environments. The climate variables used to define the three ecological groups were averaged across all localities for each species. All GIS operations were performed in ArcGIS 9.3 (ESRI, Redlands, CA, USA). The hot and dry, hot and humid, and cold environments were represented by 29, 34, and 33 species respectively.

### Experimental protocol

For all species, we measured resistance to starvation and body size for both females and males. Prior to the experiments approximately 40 pairs of parental flies were allowed to lay eggs on medium-filled spoons spread with live yeast to stimulate oviposition. From these spoons 25 eggs were collected into each of 10 vials for larval development at 20°C. Eclosing flies from different species were collected over a 3 day period and kept on holding vials for 5–8 days before they were sexed quickly under CO_2_ anaesthesia. After sexing the flies were kept in holding vials for 2 days before testing to ensure recovery from anaesthesia. This protocol was designed to ensure that all flies were non-virgin and that the age difference within and between species was ±1 and ±3 days, respectively. There was almost no mortality in this period for any of the species (or in controls held for the period that flies were stressed). For all species we assayed 10 flies of each sex for each trait. Due to the large number of species, resistance traits were tested over 6 experimental rounds. Resistance to heat, cold and desiccation were previously described [4], [21].

#### Starvation resistance

This trait was scored as the time to death in flies that were denied access to food. Individual flies were placed into 50 ml vials containing 2 ml agar to ensure that flies did not die of dehydration. Flies were scored every 12 hrs until death.

#### Body mass

Ten individuals of each sex of each species were dried at 60°C for 24 hrs before dry mass was measured to 6 decimal places using a Sartorius MC5 micro balance (Sartorius, Göttingen, Germany).

### Analysis

#### Starvation resistance and body size

Previous work [4], [21] established that heat, cold and desiccation resistance are linked to environmental variables and therefore resistance in these traits is directly related to a species' distribution. However, the extent to which the evolution of starvation resistance and body size is linked to environmental parameters has not previously been examined with this data set. Using a composite phylogeny previously published [4], we examined the extent to which environment or phylogeny shaped the evolution of starvation resistance and body size. This phylogeny is based partly on van der Linde [23] and encompassed 58 of the species considered here, with branch lengths based on genetic distances. The remaining species were incorporated into the phylogeny based on other data sets and by standardising branch lengths via a common species across the different data sets. Where branch lengths for sister species were unknown, the smallest genetic distance was used. Analyses were also repeated with branch lengths set as 1, and similar results were obtained (not presented). As multi-collinearity of environmental variables may confuse the interpretation of associations between traits and environments, we examined the single environmental variable that explained the greatest variation in traits. We did not correct the stress resistance traits for body size, because no consistent associations between size and resistance were present.

To assess the extent to which phylogeny and traits were associated, we used a number of common approaches to examine phylogenetic signal within the data set, as discussed in detail elsewhere [4]. Bloomberg's K and Pagel's λ, where traits are evolving via Brownian Motion (BM), were estimated using the picante package in R [24], [25]. λ was estimated from the residuals with significance tested by comparing the AIC_c_ ratios of the estimated λ with λ = 0 (H_0_ = no phylogenetic signal) and λ = 1 (H_a_ = phylogenetic signal). The K-statistic can be divided into four scenarios: K = 0 (H_0_ = no phylogenetic signal), K = 1 (H_a_ = phylogenetic signal), K>1 (traits are more similar than would be expected under BM), and K<1 but >0 (traits are less similar than expected under BM which may be linked to either convergent evolution of unrelated species or measurement error) [26]. Phylogenetic signal was also estimated by modelling traits as evolving under an Ornstein-Uhlenbeck process, using the SLOUCH package also implemented in R [27]. Finally, estimates of phylogenetic signal using Moran's *I* make no underlying assumptions as to the mode of evolution within traits but compute the phylogenetic autocorrelation in data at different taxonomic levels, and these were obtained using the ape package in R [28], [29].

#### Correlated trait evolution

We initially examined trait correlations across all species. Then to distinguish between correlated responses driven by genetic interactions versus correlated selection, we re-examined patterns of correlations after dividing species into the three climatic groupings outlined above.

Individual species data points are not statistically independent as species may share similarities due to evolutionary history. To consider the impact of phylogeny on trait correlations, we used the Phylogenetic Generalized Least Squares approach (PGLS), implemented in caper in R [30]. This analysis can be viewed as a special case of Felsenstein's independent contrast. However unlike independent contrasts which consider traits evolving strictly under a model of Brownian motion (evolution of traits across branches is random and independent of earlier trait states), PGLS allows for the incorporation of scaling parameters providing greater flexibility in modelling trait evolution. This may significantly improve the fit of the model and result in more accurate estimates of correlations between traits. Here we used λ, where λ reflects the level of association between phylogeny and traits, i.e. phylogenetic signal. A model of λ = 1 is equivalent to Brownian motion and thus exactly the same as independent contrasts, alternatively λ = 0 is the equivalent of a standard non-phylogenetic correlation [31]. Sexes were analysed separately because differences among sexes are well established for the stress traits as well as for size [32], and significant sex effects were detected for all traits except cold (data not shown). We also included the length of time species had been in the laboratory in initial correlations to test for effects of laboratory adaptation, but this covariate did not influence trait values or correlations (see also [4]) and is not considered further. Finally, we examined the role of body size in driving correlated trait evolution by examining the relationship between traits based on the residuals from associations between each trait and body size.

## Results

### Starvation resistance and body size, environment and phylogeny

Of the four climate variables investigated, annual mean temperature (AMT) and annual precipitation (Pann) explained the largest proportion of variation in starvation resistance for females and males respectively (females: R^2^ = 0.24, slope = −8.35 hrs/°C, P<0.001; males: R^2^ = 0.23, slope = −237.20 hrs/log mm, P<0.001) (Table S1). In contrast to starvation resistance, body size was only weakly driven by environmental variables (females: AMT, R^2^ = 0.11, slope = −0.01 mg/°C, P = 0.002; males: Pwet, R^2^ = 0.09, slope = −0.001 mg/mm, P = 0.004). Phylogeny may also play a role in shaping trait evolution. Using a number of different methods to test for phylogenetic signal (Table S2), we consistently found a moderate to strong association between phylogeny and starvation resistance and body size respectively (λ = 0 no phylogenetic signal, λ = 1 phylogenetic signal – starvation: females: λ = 0.56, males: λ = 0.73, body size: : λ = 0.92, males: λ = 1.02). Following correction for phylogeny a weaker yet significant relationship was detected for both sexes for starvation resistance (females: R^2^ = 0.10, slope = −5.50 hrs/°C, P = 0.002; males: R^2^ = 0.07, slope = −110.83 hrs/log mm, P = 0.007). The relationship between size and environment was almost entirely driven by phylogenetic relationships (females: R^2^ = 0.06, slope = −0.01 mg/°C, P = 0.005; males: R^2^ = 0.001, slope = −0.001 mg/mm, P = 0.60). Thus the evolution of starvation resistance was driven by both environment and phylogeny, with phylogeny playing a larger role in males, while for body size phylogeny had a particularly large effect.

### Correlated trait evolution

All significant correlations between resistance traits were positive such that an increase in resistance to one stress correlated with an increase in resistance to another stress (Table 1). Following correction for phylogeny, significant positive correlations were detected between cold and heat resistance, and between desiccation and resistance to cold, heat and starvation. Body size was only correlated with starvation resistance (both sexes) and weakly with cold and desiccation resistance (males only). In line with the strong phylogenetic signature we detected for body size, correlations with size disappeared after correction for phylogeny (Table 1). Moreover, correcting for body size had only small effects on the relationship between traits (Table S4a). *Drosophila mojavensis* was particularly resistant to all stressors and thus this data point could potentially drive some of the observed correlations. However, removal of this data point did not influence the overall conclusions.

**Table 1:** Correlations for resistance to desiccation (dess), cold, heat and starvation (starv), and for body size (body) for female and male *Drosophila*.

	overall females				overall males			
	cold	heat	starv	body	Cold	heat	starv	body
**dess** R^2^	**0.17**	**0.32**	**0.34**	0.04	**0.19**	**0.38**	**0.37**	**0.09**
PGLS R^2^	**0.14**	**0.17**	**0.16**	0.01	**0.13**	**0.20**	**0.18**	0.02
λ	0.72†*	0.86 †*	0.66†*	0.74†*	0.61†*	0.70†*	0.34†	0.72†*
**cold** R^2^		**0.06**	**0.12**	0.06		**0.08**	**0.15**	**0.07**
PGLS R^2^		**0.08**	0.03	0.04		**0.06**	**0.08**	0.03
λ		1*	0.99*	0.94†*		0.97*	1*	0.98*
**heat** R^2^			**0.16**	<0.01			**0.25**	0.02
PGLS R^2^			0.05	0.04			0.05	0.04
λ			1*	0.94*			0.93*	0.92*
**starv** R^2^				**0.10**				**0.25**
PGLS R^2^				<0.01				0.03
λ				0.74†*				0.79†*

Correlations are presented uncorrected and corrected for phylogeny (PGLS). Significant correlations are highlighted in bold and corrected for multiple comparisons using a sequential Bonferroni correction. Estimates of phylogenetic signal are given as λ with * indicating λ significant different from 0, while † indicates λ significant different from 1.

### Correlated trait evolution across environments

**Figure 1:**
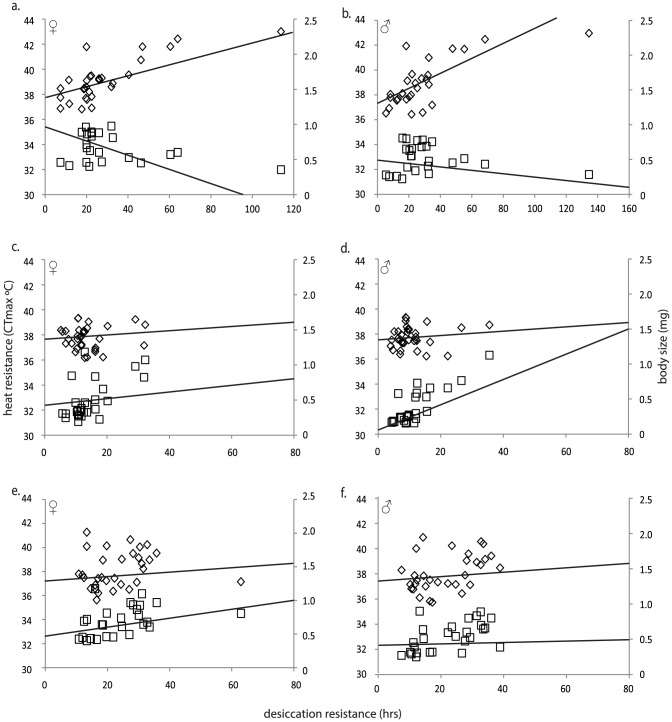
Correlations between traits across different environments. Correlations between the traits desiccation and heat resistance (◊) and desiccation resistance and body size (□) for the three environments dry and hot (a–b), hot and wet (c–d) and cold (e–f). The solid line represents the correlation between traits following correction for phylogeny.

Here we split species into three different environments - hot and dry, hot and wet and cold. We ignore the effects of size on correlations between stress resistance traits because size-corrected correlations did not differ much from uncorrected correlations (Table S4 b & c). Trait correlations differed across all three environments. Under hot and dry environments, all traits except body size associated strongly with Pann (Table 2) and significant correlations between all resistance traits were detected. In contrast, trait correlations observed in hot and dry environments were absent from the hot and wet environments, particularly the correlations between resistance to desiccation and to the other stresses (Tables 3 and 4, Figure 1). Furthermore, there was no evidence of an association between environment and traits (Table 2). Removal of single data points that could be considered outliers (Figure 1) for the hot and dry and cold environments did not influence these conclusions. Desiccation resistance was found to correlate with body size but only in males following correction for phylogeny. Species occupying hot and wet environments also tended to be less stress resistant than species occupying hot and dry environments (Table S3).

**Table 2:** Relationship between environmental variables and stress traits, desiccation (dess), heat, cold and starvation (starv) resistance as well as body size (body) with species divided into the three environments: warm and dry, warm and wet, and cold.

	hot and dry		hot and wet		cold	
	Pann ♀	Pann ♂	Pann ♀	AMT ♂	AMT ♀	AMT ♂
**Dess -** R^2^	**0.76**	**0.74**	**0.13**	0.03	**0.18**	**0.25**
PGLS R^2^	**0.76**	**0.74**	0.01	0.001	**0.14**	**0.13**
λ	0†	0†	0.95*	0.99*	0.29†	0.87*
**heat -** R^2^	**0.53**	**0.51**	0.001	0.03	0.001	0.001
PGLS R^2^	**0.53**	**0.51**	0.001	0.001	0.06	0.01
λ	0†	0†	0.76*	0.40†	0.85*	0.84*
**cold -** R^2^	**0.46**	**0.49**	**0.10**	0.06	**0.27**	**0.24**
PGLS R^2^	**0.46**	**0.49**	0.12	0.001	**0.24**	0.20
λ	0†	0†	0.72	0.97*	0.76*	0.82*
**starv -** R^2^	**0.46**	**0.46**	0.001	**0.19**	**0.26**	**0.36**
PGLS R^2^	**0.46**	**0.46**	0.10*	0.001	**0.23**	**0.27**
λ	0†	0†	0.97*	0.95*	0.54*	0.64*†
**body -** R^2^	0.01	0.06	0.001	**0.13**	0.06	0.06
PGLS R^2^	0.06	0.10*	0.001	0.001	0.001	0.001
λ	0.99*	1*	1*	1*	0.81*	0.92*

The environmental variables (annual precipitation = Pann and annual mean temperature = AMT) that explained the greatest variation for traits are presented. Significant correlations are highlighted in bold with associations between traits and environmental variables presented both uncorrected and corrected for phylogeny (PGLS). Estimates of phylogenetic signal are given as λ with * indicating λ significant different from 0, while † indicates λ significant different from 1.

**Table 3:** Correlated responses for resistance to desiccation (dess), cold, heat, and starvation (starv) and body size (body) for female *Drosophila*, divided into three environments (hot and dry, hot and wet, cold).

	hot and dry				hot and wet				cold			
Females	cold	heat	starv	body	cold	heat	starv	body	cold	heat	starv	body
**dess** R^2^	**0.46**	**0.60**	**0.41**	0.11	0.14	0.001	0.03	**0.38**	0.02	0.02	**0.47**	0.16
	±0.17	±0.14	±0.15	±0.11	±0.12	±0.05	±0.07	±0.19	±0.06	±0.05	±0.14	±0.13
PGLS R^2^	**0.39**	**0.60**	**0.22**	**0.17**	0.09	0.001	0.001	0.05	0.03	0.002	**0.47**	0.16
λ	0.51**†**	0**†**	0.59**†**	0.87*	0.93*	0.96*	1*	0.92	0.44**†**	0.42**†**	0**†**	0**†**
**cold** R^2^		**0.36**	**0.43**	0.01		0.001	0.04	0.005		0.04	0.005	0.002
		±0.19	±0.14	±0.09		±0.04	±0.06	±0.06		±0.09	±0.09	±0.06
PGLS R^2^		**0.27**	**0.41**	0.001		0.04	0.08	0.03		0.001	0.005	0.002
λ		0.40**†**	0**†**	**0†**		0.81*	0.81	0.54*		0.83*	0.96*	0.92*
**heat** R^2^			**0.51**	0.11			0.009	0.04			0.08	0.11
			±0.14	±0.13			±0.06	±0.10			±0.11	±0.13
PGLS R^2^			**0.49**	**0.23**			0.001	0.001			0.02	0.001
λ			0**†**	0.83*			0.69*†	0.66			1*	1*
**starv** R^2^				0.09				0.02				0.28
				±0.10				±0.08				±0.15
PGLS R^2^				0.001				0.11				**0.28**
λ				0.85*				0.92				0**†**

Correlations are presented uncorrected and corrected for phylogeny (PGLS). Significance is highlighted in bold with correlations corrected for multiple comparisons using a sequential Bonferroni approach. Estimates of phylogenetic signal are given as λ with * indicating λ significant different from 0, while † indicates λ significant different from 1.

**Table 4:** Correlated responses for resistance to desiccation (dess), cold, heat, and starvation resistance (starv), and body size (body) for male *Drosophila*, divided into three environments (hot and dry, hot and wet, cold).

	hot and dry				hot and wet				cold			
males	cold	heat	starv	body	cold	heat	starv	body	cold	heat	starv	body
**dess** R^2^	**0.54**	**0.51**	**0.51**	0.02	0.14	0.02	0.18	**0.70**	0.21	0.19	**0.38**	**0.30**
	±0.12	±0.14	±0.12	±0.07	±0.10	±0.05	±0.10	±0.17	±0.11	±0.13	±0.14	±0.18
PGLS R^2^	**0.53**	**0.49**	**0.49**	0.02	0.09	0.02	0.04	**0.58**	**0.39**	0.04	0.001	0.001
λ	0**†**	0**†**	0**†**	0.66	0.97*	0.97*	1*	0.80*†	0.95*	0.97*	1*	1*
**cold** R^2^		**0.44**	**0.41**	0.001		0.001	0.04	0.01		0.09	0.07	0.02
		±0.18	±0.18	±0.05		±0.06	±0.07	±0.06		±0.10	±0.10	±0.08
PGLS R^2^		**0.33**	**0.39**	0.001		0.01	0.007	0.03		0.04	0.08	0.001
λ		0.52**†**	0**†**	0.60		0.44**†**	0.83*	0.77*		0.72*†	0.99*	0.94*
**heat** R^2^			**0.55**	0.03			0.03	0.002			0.15	0.15
			±0.15	±0.08			±0.06	±0.07			±0.13	±0.16
PGLS R^2^			**0.53**	0.05			0.005	0.001			0.02	0.001
λ			0**†**	0.83*			0.48**†**	0**†**			0.96*	0.92
**starv** R^2^				0.001				0.31				**0.47**
				±0.04				±0.14				±0.14
PGLS R^2^				0.001				0.01				**0.47**
λ				0.89*				0.93				0**†**

Correlations are presented uncorrected and corrected for phylogeny (PGLS). Significance is highlighted in bold, with correlations corrected for multiple comparisons using a sequential Bonferroni approach. Estimates of phylogenetic signal are given as λ with * indicating λ significant different from 0, while † indicates λ significant different from 1.

In cold environments, cold resistance did not correlate with any trait in females, while in males cold resistance correlated with desiccation resistance (Table 3 and 4). The only other trait correlations in cold environments were between starvation resistance and body size for both sexes and between starvation and desiccation resistance in females only. These traits were also found to significantly associate with the environmental variable AMT (Table 2).

## Discussion

Previous work has shown that environmental conditions have shaped the evolution of cold, desiccation and heat resistance in species of *Drosophila* [4], [21] and in other insects [33]–[35]. Consequently these traits are likely to be important for predicting species responses to climate change, particularly when there are strong phylogenetic constraints acting on them [4], [21], [36]. Here we have extended this work by focussing not just on the traits themselves but also on interactions among them as well as with starvation resistance and body size. We have considered these additional traits because they are often genetically correlated with climatic stress resistance at the intraspecific level [32] and because the evolution of size and starvation may be driven by climatic adaptation as suggested by clinal patterns at the intra-specific level [37], [38].

We initially found that both phylogeny and environment played a role in shaping the evolution of starvation resistance, while the evolution of body size was primarily driven by evolutionary history. We then examined trait correlations across all species and found, in contrast to previous work [10], [15], [16], all correlations were positive such that no trade-offs were detected between any of the stress traits or body size. By examining correlated evolution of stress resistance across different environments we can begin to examine the role of trait correlations in limiting the direction of evolution. We hypothesise that if correlations change depending on the environment, then trait combinations are free to evolve and the evolution of stress resistance is not constrained by trait correlations. Here we found hot and dry environments selected for correlated trait evolution across most traits, except body size. This was coupled with on average higher trait means in comparison to hot and wet and to cold environments, as well as a strong relationship between stress traits and the environment. This combined set of results suggests selection is driving the evolution of these traits in hot and dry environments. In contrast, very few traits were correlated in hot and wet and cold environments and only weak associations between traits and the environment. Patterns of correlated trait evolution across the environments fit with our a priori hypotheses on the direction of correlated evolution driven by correlated selection.

Under hot and dry environments we anticipated selection will be strongest for heat and desiccation stress, particularly as the opportunity for behavioural thermoregulation is likely to be low in *Drosophila* [39]. We also anticipated a positive relationship between cold and heat resistance in these environments, contrary to the hypothesised cold/heat trade-off [10], [40], primarily because many of the hot and dry species can be defined as xeric cactophilic species. These environments fluctuate greatly with temperatures inside cactus rots (may offer a thermal refuge) fluctuating from 5 to 40°C within a day [41]. In line with these predicted selection pressures, we found positive associations between these stress traits as well as positive associations with starvation resistance (Table 3 and 4, Figure 1). These results further support our hypothesis that thermal breadth is driven by selection in these environments and is unlikely to be linked to genetic/mechanistic factors and are in contrast to intra-specific patterns [10], [40].

Similar to hot and dry environments the relationship between traits in hot and wet and cold environments fit with the hypothesis of correlated selection. Under hot and wet environments we anticipated selection for desiccation and cold resistance to be weak. Furthermore, in these environments the opportunity for behavioural thermoregulation is expected to be higher because the greater canopy cover in wet environments provides microenvironments where flies can evade stressful conditions and thus avoid strong selection on heat resistance [42], [43]. As expected from these predictions, species occupying these environments were not very stress resistant and no significant correlations were detected between the stress traits.

Similarly under cold environments there was an expectation of relaxed selection for heat resistance (Table S3). Consistent with this expectation we found no relationship between heat and cold resistance (Table 3 and 4). However cold, desiccation and starvation resistance did correlate in at least one sex following correction. Given that cold environments are often dry and that desiccation resistance is correlated to precipitation in species comparisons, an association between cold and desiccation resistance may not unexpected. However, neither cold nor desiccation resistance was found to associate with any precipitation variables or our measure of aridity (dpa) (data not shown). Further work establishing what is driving these patterns is needed. The expected pattern of selection on starvation resistance is however unclear; resources might be scarcer or more sporadic in cold environments. However some intraspecific studies suggest selection for starvation resistance in tropical rather than temperate environments [38], [44]. Desiccation and starvation resistance have also frequently been positively associated both at the intra- and inter-specific level, leading to suggestions that similar mechanisms/genes underlie these traits [16], [17]. Body size has also been commonly linked to stress traits [19], [45],

Although most patterns of correlated traits were found to be consistent with correlated evolution, we found some examples that could reflect genetic/mechanistic interactions. We suspect that the significant correlation between male body size and desiccation resistance in hot and wet environments may reflect a genetic/mechanistic interaction rather than a correlated pattern of selection. Body size has often been implicated in the evolution of desiccation resistance because larger body size may reduce relative water-loss rate through a reduced surface/volume relationship [3], [46]. However, the weak association in males and contrasting associations in females between these traits in hot and dry environments suggest that, even when some constraints underpin trait associations, they can be decoupled from trait evolution when selection for tolerance is present (Figure 1). It is also possible that the correlation between body size and starvation resistance in cold environments reflects genetic/mechanistic association because larger body size may allow for greater storage of lipids and/or also reduce mass specific energetic turnover through the overall relationship between mass specific metabolic rate and size, which is core to the metabolic theory of ecology [47], [48].

In summary, these data suggest that associations involving stress resistance traits are mostly driven by selection rather than genetic/mechanistic constraints. A lack of consistent trait associations across different environments suggests that, even when genetic interactions occur, they can be broken down by selection. Some studies have demonstrated that trait correlations may limit/slow future evolutionary responses [9], [49], but our results agree with others [50], [51] who argue that genetic correlations rarely influenced adaptive potential.

## Supporting Information

Table S1 **Regression analysis for the association between environmental variables, starvation resistance and body size.** Relationship between starvation resistance (starv) and body size for latitude and seven environmental variables; annual mean temperature (amt), maximum temperature of the warmest month (T_max_), temperature seasonality (Tsea), log annual precipitation (Pann). precipitation of the driest month (Pdry), precipitation of the wettest month (Pwet) and precipitation seasonality (Psea).A multiple regression approach was taken to examine the relationship between starvation and body size and multiple environmental variables. The explanatory power of both the single and multiple predictor models is given. Significant *P* values and the model with the best Akaike Information Criterion (AIC) are highlighted in bold.
(DOCX)

Table S2 **Estimates of phylogenetic signal for starvation resistance and body size.** Phylogenetic signal was assessed through alternative methods. λ and K range from no phylogenetic signal λ and K = 0 to high phylogenetic signal with λ = 1, K≥1. Significance of λ is tested against a model where λ = 0 and λ = 1 the estimate with the best corrected Akaike Information Criterion (AICc) is given in bold. The SLOUCH method estimates phylogenetic signal by fitting an *Ornstein-Uhlenbeck* (*OU*) *model*. Phylogenetic signal is estimated through the t half-life (t_1/2_) where a t_1/2_>0 reflects an increasing association between the phylogeny and the trait (t_1/2_ has the same units as the phylogeny, here tree height = 1). Moran's *I* provides an estimate of the autocorrelation found within a dataset at three taxonomic levels: subgenus (SubG), species group (SppG) and subspecies group (SubSppG). * significance at the P = 0.05 level, ** P = 0.01, ***P<0.001.
(DOCX)

Table S3 **Trait means for stress traits and body size under the three environments.** Species mean resistance for traits desiccation, cold, heat and starvation resistance as well as body size for the three defined environments hot and dry, hot and wet and cold.
(DOCX)

Table S4 **Trait correlations following correction for body size.** Traits were corrected for body size a) across all species b) across the three environments for females and c) across the three environments for males. Values highlighted in bold were significant following sequential bonferroni correction.
(DOCX)
