# Supplementary material for: Trait Associations across Evolutionary Time within a Drosophila Phylogeny: Correlated Selection or Genetic Constraint?
Source: PLoS One. 2013 Aug 28;8(8):e72072. doi: 10.1371/journal.pone.0072072 (PMC3756044; doi:10.1371/journal.pone.0072072)
Supplement: Table S1 — Regression analysis for the association between environmental variables, starvation resistance and body size. Relationship between starvation resistance (starv) and body size for latitude and seven environmental variables; annual mean temperature (amt), maximum temperature of the warmest month (Tmax), temperature seasonality (Tsea), log annual precipitation (Pann). precipitation of the driest month (Pdry), precipitation of the wettest month (Pwet) and precipitation seasonality (Psea).A multiple regression approach was taken to examine the relationship between starvation and body size and multiple environmental variables. The explanatory power of both the single and multiple predictor models is given. Significant P values and the model with the best Akaike Information Criterion (AIC) are highlighted in bold. (DOCX) [file pone.0072072.s001.docx]

Table S1. **Regression analysis for the association between environmental variables, starvation resistance and body size**

Relationship between starvation resistance (starv) and body size for latitude and seven environmental variables; annual mean temperature (amt), maximum temperature of the warmest month (T_max_), temperature seasonality (Tsea), log annual precipitation (Pann). precipitation of the driest month (Pdry), precipitation of the wettest month (Pwet) and precipitation seasonality (Psea).A multiple regression approach was taken to examine the relationship between starvation and body size and multiple environmental variables. The explanatory power of both the single and multiple predictor models is given. Significant *P* values and the model with the best Akaike Information Criterion (AIC) are highlighted in bold.

|  | **Females** |  |  |  | **males** |  |  |  |
| --- | --- | --- | --- | --- | --- | --- | --- | --- |
|  | **R^2^** | **slope** | **P** | **AIC** | **R^2^** | **slope** | **P** | **AIC** |
| **Starvation** |  |  |  |  |  |  |  |  |
| AMT | **0.24** | **-8.54** | **<0.001** | **804.47** | 0.21 | -6.10 | <0.001 | 773.21 |
| Lat | 0.21 | 3.45 | <0.001 | 806.70 | 0.18 | 2.51 | <0.001 | 775.36 |
| Tmax | 0.20 | -15.52 | <0.001 | 807.57 | 0.15 | -10.68 | <0.001 | 777.57 |
| Tsea | 0.16 | 0.16 | <0.001 | 810.99 | 0.14 | 0.11 | 0.001 | 778.88 |
| Pann | 0.18 | -275.27 | <0.001 | 809.04 | **0.23** | **-237.20** | **<0.001** | **771.30** |
| Pdry | 0.01 | 0.35 | 0.63 | 823.66 | 0.01 | -0.04 | 0.94 | 789.96 |
| Pwet | 0.19 | -0.42 | <0.001 | 808.83 | 0.21 | -0.35 | <0.001 | 772.79 |
| Psea | 0.12 | -1.77 | 0.003 | 814.45 | 0.08 | -1.17 | 0.01 | 783.35 |
| dpa | <0.01 | 0.73 | 0.38 | 823.12 | 0.02 | 2.46 | 0.11 | 787.33 |
| AMT + lat | 0.23 | - | <0.001 | 806.41 | - |  |  |  |
| Pann+ Pwet | - | - |  | - | 0.23 | - | <0.001 | 772.17 |
| **Body size** |  |  |  |  |  |  |  |  |
| AMT | **0.11** | **0.89** | **0.002** | **0.86** | 0.08 | 0.69 | 0.006 | -13.87 |
| Lat | 0.07 | 0.49 | 0.01 | 4.13 | 0.05 | 0.36 | 0.02 | -11.35 |
| Tmax | 0.06 | 1.27 | 0.02 | 5.11 | 0.03 | 1.03 | 0.03 | -11.19 |
| Tsea | 0.09 | 0.49 | 0.004 | 2.05 | 0.07 | 0.001 | 0.01 | -12.44 |
| Pann | 0.05 | 1.86 | 0.02 | 5.25 | 0.03 | 1.40 | 0.05 | -9.90 |
| Pdry | 0.01 | 0.59 | 0.74 | 10.64 | 0.001 | 0.44 | 0.53 | -6.48 |
| Pwet | 0.09 | 0.79 | 0.004 | 2.61 | **0.08** | **0.63** | **0.006** | **-13.94** |
| Psea | 0.01 | 0.73 | 0.17 | 8.78 | 0.02 | 0.11 | 0.56 | -8.69 |
| dpa | 0.01 | 0.50 | 0.20 | 9.04 | <0.01 | 0.38 | 0.30 | -7.20 |
| AMT + lat | 0.10 | - | - | 2.77 | - | - | - | - |
| Pann+ Pwet | - | - | - | - | 0.09 | - | - | -13.51 |
