# Supplementary material for: Trait Associations across Evolutionary Time within a Drosophila Phylogeny: Correlated Selection or Genetic Constraint?
Source: PLoS One. 2013 Aug 28;8(8):e72072. doi: 10.1371/journal.pone.0072072 (PMC3756044; doi:10.1371/journal.pone.0072072)
Supplement: Table S2 — Estimates of phylogenetic signal for starvation resistance and body size. Phylogenetic signal was assessed through alternative methods. λ and K range from no phylogenetic signal λ and K = 0 to high phylogenetic signal with λ = 1, K≥1. Significance of λ is tested against a model where λ = 0 and λ = 1 the estimate with the best corrected Akaike Information Criterion (AICc) is given in bold. The SLOUCH method estimates phylogenetic signal by fitting an Ornstein-Uhlenbeck (OU) model. Phylogenetic signal is estimated through the t half-life (t1/2) where a t1/2>0 reflects an increasing association between the phylogeny and the trait (t1/2 has the same units as the phylogeny, here tree height = 1). Moran's I provides an estimate of the autocorrelation found within a dataset at three taxonomic levels: subgenus (SubG), species group (SppG) and subspecies group (SubSppG). * significance at the P = 0.05 level, ** P = 0.01, ***P<0.001. (DOCX) [file pone.0072072.s002.docx]

Table S2. **Estimates of phylogenetic signal for starvation resistance and body size**. Phylogenetic signal was assessed through alternative methods. λ and K range from no phylogenetic signal λ and K = 0 to high phylogenetic signal with λ = 1, K ≥ 1. Significance of λ is tested against a model where λ=0 and λ=1 the estimate with the best corrected Akaike Information Criterion (AICc) is given in bold. The SLOUCH method estimates phylogenetic signal by fitting an Ornstein-Uhlenbeck (OU) model. Phylogenetic signal is estimated through the t half-life (t_1/2_) where a t_1/2_ > 0 reflects an increasing association between the phylogeny and the trait (t_1/2_ has the same units as the phylogeny, here tree height =1). Moran’s *I* provides an estimate of the autocorrelation found within a dataset at three taxonomic levels: subgenus (SubG), species group (SppG) and subspecies group (SubSppG). * significance at the P=0.05 level, ** P=0.01, ***P<0.001

|  | λ | AICc | K | SLOUCH t_1/2_ | Morans *I* | | |
| --- | --- | --- | --- | --- | --- | --- | --- |
|  |  |  |  |  | SubG | SppG | SubSppG |
| starv: ♀ | 0.56 | **795.65** | 0.55*** | 0.45 (0.22 - 1.80) | 0.53*** | 0.40*** | 0.20*** |
|  |  | λ _1_ 806.83 |  |  |  |  |  |
|  |  | λ _0_ 804.47 |  |  |  |  |  |
| starv: ♂ | 0.73 | **753.47** | 0.70*** | 0.64 (0.30 - ∞) | 0.51*** | 0.50*** | 0.22*** |
|  |  | λ _1_ 755.16 |  |  |  |  |  |
|  |  | λ _0_ 771.30 |  |  |  |  |  |
| body: ♀ | 0.92 | -60.97 | 1.04*** | ∞ (2.80 - ∞) | 0.60*** | 0.58*** | 0.45*** |
|  |  | λ _1_-61.69 |  |  |  |  |  |
|  |  | λ _0_-0.06 |  |  |  |  |  |
| body: ♂ | 1.02 | -106.02 | 1.54*** | ∞ (40 - ∞) | 0.72*** | 0.67*** | 0.54*** |
|  |  | λ _1_-107.53 |  |  |  |  |  |
|  |  | λ _0_-14.78 |  |  |  |  |  |
