# Supplementary material for: Trait Associations across Evolutionary Time within a Drosophila Phylogeny: Correlated Selection or Genetic Constraint?
Source: PLoS One. 2013 Aug 28;8(8):e72072. doi: 10.1371/journal.pone.0072072 (PMC3756044; doi:10.1371/journal.pone.0072072)
Supplement: Table S3 — Trait means for stress traits and body size under the three environments. Species mean resistance for traits desiccation, cold, heat and starvation resistance as well as body size for the three defined environments hot and dry, hot and wet and cold. (DOCX) [file pone.0072072.s003.docx]

Table S3. **Trait means for stress traits and body size under the three environments**

Species mean resistance for traits desiccation, cold, heat and starvation resistance as well as body size for the three defined environments hot and dry, hot and wet and cold.

|  | **Hot and dry** | **Hot and wet** | **Cold** |
| --- | --- | --- | --- |
| **Females** |  |  |  |
| Desiccation | 29.27 ± 4.11 | 13.46 ± 1.10 | 23.55 ± 1.86 |
| Cold | 4.78 ± 0.30 | 6.53 ± 0.21 | 2.23 ± 0.39 |
| Heat | 39.13 ± 0.32 | 37.79 ± 0.15 | 38.18 ± 0.26 |
| Starvation | 228.65 ± 16.05 | 167.40 ± 9.60 | 242.53 ± 23.01 |
| Body size | 0.66 ± 0.04 | 0.49 ± 0.05 | 0.70 ± 0.04 |
| **Males** |  |  |  |
| Desiccation | 28.83 ± 4.74 | 10.86 ± 1.11 | 21.43 ± 1.67 |
| Cold | 4.60 ± 0.26 | 6.62 ± 0.21 | 2.03 ± 0.41 |
| Heat | 39.13 ± 0.35 | 37.79 ± 0.14 | 38.19 ± 0.25 |
| Starvation | 177.63 ± 13.83 | 127.86 ± 9.12 | 185.64 ± 17.09 |
| Body size | 0.52 ± 0.04 | 0.37 ± 0.05 | 0.53 ± 0.04 |
