# Supplementary material for: Trait Associations across Evolutionary Time within a Drosophila Phylogeny: Correlated Selection or Genetic Constraint?
Source: PLoS One. 2013 Aug 28;8(8):e72072. doi: 10.1371/journal.pone.0072072 (PMC3756044; doi:10.1371/journal.pone.0072072)
Supplement: Table S4 — Trait correlations following correction for body size. Traits were corrected for body size a) across all species b) across the three environments for females and c) across the three environments for males. Values highlighted in bold were significant following sequential bonferroni correction. (DOCX) [file pone.0072072.s004.docx]

Table S4. **Trait correlations following correction for body size**

Traits were corrected for body size a) across all species b) across the three environments for females and c) across the three environments for males. Values highlighted in bold were significant following sequential bonferroni correction.

a)

| **overall females** | | | | **overall males** | | |
| --- | --- | --- | --- | --- | --- | --- |
|  | cold | heat | starv | cold | heat | starv |
| **dess**  R^2^ | **0.14** | **0.21** | **0.31** | **0.24** | **0.29** | **0.37** |
| bs R^2^ | **0.09** | **0.21** | **0.23** | **0.17** | **0.29** | **0.15** |
| **cold** R^2^ |  | 0.06 | **0.09** |  | **0.07** | **0.13** |
| bs R^2^ |  | 0.05 | 0.04 |  | 0.05 | 0.04 |
| **heat** R^2^ |  |  | **0.07** |  |  | **0.11** |
| bs R^2^ |  |  | 0.06 |  |  | **0.08** |

b)

| **hot and dry** | | | | **hot and wet** | | | **cold** | | |
| --- | --- | --- | --- | --- | --- | --- | --- | --- | --- |
| Females | cold | heat | starv | cold | heat | starv | cold | heat | starv |
| **dess**  R^2^ | **0.26** | **0.57** | 0.17 | 0.07 | <0.01 | <0.01 | <0.01 | <0.01 | **0.43** |
| bs R^2^ | **0.33** | **0.60** | 0.24 | 0.12 | 0.05 | <0.01 | <0.01 | <0.01 | **0.33** |
| **cold** R^2^ |  | 0.12 | 0.19 |  | <0.01 | 0.01 |  | 0.07 | <0.01 |
| bs R^2^ |  | 0.21 | **0.26** |  | <0.01 | <0.01 |  | 0.03 | <0.01 |
| **heat** R^2^ |  |  | **0.27** |  |  | <0.01 |  |  | 0.01 |
| bs R^2^ |  |  | **0.29** |  |  | <0.01 |  |  | 0.03 |

c)

| **hot and dry** | | | | **hot and wet** | | | **cold** | | |
| --- | --- | --- | --- | --- | --- | --- | --- | --- | --- |
| Females | cold | heat | starv | cold | heat | starv | cold | heat | starv |
| **dess**  R^2^ | **0.46** | **0.39** | 0.22 | 0.05 | <0.01 | 0.10 | **0.22** | 0.13 | **0.43** |
| bs R^2^ | **0.47** | **0.44** | 0.21 | 0.19 | 0.09 | <0.01 | **0.24** | <0.01 | 0.18 |
| **cold** R^2^ |  | **0.24** | 0.07 |  | <0.01 | 0.11 |  | 0.10 | 0.05 |
| bs R^2^ |  | **0.28** | 0.18 |  | <0.01 | 0.01 |  | 0.05 | <0.01 |
| **heat**  R^2^ |  |  | 0.23 |  |  | <0.01 |  |  | 0.08 |
| bs R^2^ |  |  | **0.25** |  |  | <0.01 |  |  | <0.01 |
